# Why Children Believe They Are Owned

**DOI:** 10.1162/opmi_a_00090

**Published:** 2023-08-01

**Authors:** Christina Starmans, Ori Friedman

**Affiliations:** Department of Psychology, University of Toronto, Toronto, Canada; Department of Psychology, University of Waterloo, Waterloo, Canada

**Keywords:** ownership, autonomy, development

## Abstract

Owners decide what happens to their property, and so adults typically view autonomous beings as non-owned. If children likewise consider autonomy when judging what is owned, this may have implications for how they view themselves. If children believe that parents have power over them, that they themselves lack autonomy, and that only the autonomous cannot be owned, this may lead them to believe that they are owned by their parents. Across three experiments, we found that 4- to 7-year-old children (*N* = 206) consistently affirm that children are owned by their parents. In Experiment 1, children judged that children and domesticated animals are owned, but denied this for adults and wild animals. In Experiment 2, children were more likely to see children as owned by their parents than by their teachers, and also denied that children own either kind of adult. Finally, in Experiment 3, children were less likely to view a child who makes decisions against parental authority as owned. These judgments are unlikely to mirror what children have been told. Instead, they likely result from children spontaneously using autonomy principles, and possibly other principles of ownership, in reasoning about the ownership of living entities.

## INTRODUCTION

What sorts of things can be owned? Human-made artifacts—iPhones and rain boots and station wagons—make up the vast majority of items owned by members of modern society. Natural kinds, like fossilized rocks, twigs, and apples, can also be owned. And most living things can be owned. Plants, trees, and the land they occupy have been central to claims of ownership since the invention of agriculture. Keeping dogs as pets dates to at least the Paleolithic era (Janssens et al., 2018), and today people own animals for many reasons: as pets, livestock, for breeding, and in zoos. Yet in modern society, we do not view human beings as the sort of thing that can be owned, and abhor anyone who attempts to do so.

One reason for this may be that humans are seen as autonomous—as having the right and ability to make decisions for themselves. Autonomy is relevant to how we think about responsibility (Feltz & Cova, 2014), personal rights (Nucci & Lee, 1993), consent (Demaree-Cotton & Sommers, 2022)—and ownership. Because owners decide what happens to their property, a fully autonomous being cannot be owned. Yet, historically, and in some hidden parts of the world today, humans have been enslaved, and may have been viewed by some as owned. People have also been seen as property in other contexts. For instance, many cultures have seen children and women as property (e.g., Shanahan, 2007). However, how can the claim that autonomous beings cannot be owned be reconciled with the fact that humans have at least sometimes been viewed as owned by other humans? One possibility is that certain groups, or certain individuals, maybe be viewed by other groups as inherently less autonomous. Aristotle (350 BCE/1885), for instance, argued that some individuals, though human, are natural slaves, incapable of self-governance. Likewise, plantation owners in the American South would often explicitly justify the practice of slavery by claiming that enslaved peoples were “unfit for freedom” (Wise, 2001, p. 17).^1^

Although there are many historical and psychological factors involved in the history of humans being seen and treated as property, the views described above suggest that attributions of autonomy may play an important role in intuitions about when humans (and perhaps other living beings) may be thought of as owned (see also Starmans, 2023). Recent research has also tested this possibility empirically, and found that American adults’ judgments of whether a human was owned depended on whether he was described as autonomous—that is, having the ability to make decisions for himself, to decide whether to act on his desires or follow others’ instructions, and being typically held responsible for his actions. However, other important human qualities, such as sophisticated intelligence, the ability to experience a wide range of emotions, or the ability to communicate with language, did not affect ownership judgments (Starmans & Friedman, 2016). Interestingly, viewing someone as lacking autonomy did not impact judgments pertaining to the (im)morality of attempting to own them, and only affected judgments of whether they were indeed owned.

If a perceived lack of autonomy leads to a human being viewed as more ownable, this raises interesting questions about how human children are viewed. Very young children lack the capacity to make decisions for themselves, and even older children are not typically granted this right. And children themselves believe that their parents’ authority over them is legitimate and rational, not the product of unjust coercion (Braine et al., 1991). As such, autonomy-based reasoning might lead to the conclusion that children are owned by their parents.

Adults show some signs of reasoning this way. Starmans and Friedman (2016) describe a scenario in which a man sells either himself, his child, or an unrelated man to another person. When asked whether the person in question was now owned by the purchaser, adults denied that the unrelated man was owned, but agreed that the man himself (who autonomously chose to give up his freedom) was owned. Interestingly, judgments of whether the child was owned fell between these two judgments, suggesting that adults were uncertain whether a father could legitimately sell his son. One possible reason for these findings is that adults may implicitly view children as owned by their parents, but may be disinclined to describe children as being owned, because of a broad societal taboo forbidding the ownership of any human (Fiske & Tetlock, 1997).

Children themselves, however, may be less concerned about the taboo, perhaps because they have had less exposure to this rule or perhaps because they are less inclined than adults to accord humans special moral worth (McGuire et al., 2023; Sommer et al., 2019; Wilks et al., 2021). This suggests that children’s judgments may provide a way to explore whether autonomy-based reasoning leads to a perception of human children as owned by their parents.

There is some evidence that principles of autonomy and control do infuse children’s views about ownership (for a recent review see Pesowski et al., 2022). Young children, and even toddlers, are selective about which kinds of things can be owners—for example, they think robots can own things, but deny ownership to inanimate objects (e.g., Golinkoff & Markessini, 1980; Huh & Friedman, 2017; Kanngiesser et al., 2015; Noles et al., 2012). Indeed, children’s notion of *owner* may be narrower than that of adults—5- and 6-year-olds judge that a person who is in a coma, asleep, or tied up no longer owns things (Noles et al., 2012). Perhaps children believe that because people in these states cannot actively control objects, they cannot own them.

Other studies suggest that children are also sensitive to autonomy in their judgments about what can be owned. Children normally judge that people own artifacts and natural kinds in their backyards (Goulding & Friedman, 2018). However, if an animal is in someone’s yard, 5- to 8-year-olds view it as owned only if it is caged or trapped, and not if it can freely escape (Espinosa & Starmans, 2020). More generally, children have some appreciation of the rights that autonomy bestows. Four- to seven-year-olds believe that touching someone’s property or body is a greater violation than simply looking at the property or person, and view the acceptability of physical contact as dependent on the owner’s consent (Van de Vondervoort & Friedman, 2015; Van de Vondervoort et al., 2017; for related discussion see Gelman et al., 2021).

If children believe that parents have power over them, that they themselves lack autonomy, and that only autonomous beings cannot be owned, this may lead them to believe that they are owned by their parents. Such a finding would be of considerable intuitive interest and would also bear on theoretical debates about the cognitive systems engaged in understanding ownership (e.g., Boyer, 2022; Morewedge, 2021; Nancekivell et al., 2019). We explore this in three studies.

The studies reported in this article were not preregistered. The data and materials for all studies are publicly accessible at https://osf.io/gxmkd/.

## EXPERIMENT 1

Our main research question is whether children view themselves as owned by their parents. Because little research has investigated either children’s or adults’ judgments about the ownership of living beings, Experiment 1 began by exploring how both age groups judge the ownership status of two ontological kinds (humans and animals), each with two levels of autonomy (adults vs. children, and wild vs. domesticated animals).

### Method

#### Participants.

We tested 101 American adults (19–75 years, mean = 38 years, 38% female) using Amazon Mechanical Turk using the CloudResearch toolkit for quality control (Litman et al., 2017). Participants reported themselves to be 66% White, 10% Black, 9% Asian, 6% Hispanic, and 9% two or more races. We also recruited 104 children aged 4 to 7 years (mean age = 68 months, 48% female). The sample size was initially set at 20 children per age, for a total of 80. However, we ended up testing quite a few more children due to multiple experimenters testing in different locations. In all experiments, children were recruited in-person through lab databases, local preschools, and a local museum. We did not collect specific demographic data for children, however the city population we sampled is estimated to be comprised of the following ethnic groups: 44% White, 34% Black, 5% Asian, and 17% other or two or more races (United States Census Bureau). A further 14 children were tested but were excluded for having a “Yes”-bias (10) or a “No”-bias (2) in their answers to the warmup questions (see below), or for parental interference (2). No adults showed a yes-bias or no-bias in their response to warm-up questions, thus none were excluded.

#### Procedure.

Children were initially told the activity would involve questions about owning things. To provide a rough definition of ownership, and to ensure that children could understand and use the word “own”, they were asked: “Do you know what it means to own something? It means that you have it and it belongs to you. For example, I own this computer. What is something that you own?” (modeled on Noles & Gelman, 2014). If the child did not provide an answer that was easily identifiable as property, they were asked a follow-up question: “Can you tell me something else that you own? Do you own any toys?” All children provided a reasonable answer after the second question. Children were next introduced to a puppet who did not know which kinds of things people could own, but would like to find out. Children were asked to tell the puppet whether anyone owned a particular entity: “So Henry wants to know—does anyone own this ______?” Although some studies have used other questions to ask about ownership, such as “Does this ____ belong to anyone?”, in the context of living beings we worried that children might see other ways of interpreting these questions (e.g., children might be thought to “belong” to their family, without a connotation of ownership). To avoid this, we asked children directly about ownership, building on other recent work that provides evidence that children are able to understand and use the term “own” correctly (e.g., Banerjee et al., 2015; Nancekivell et al., 2023; Noles et al., 2012; Noles & Gelman, 2014; Shaw et al., 2012).

In the first phase of the study, children answered six warm-up questions about artifacts (ball, chair), natural kinds (pinecone, leaf), and un-owned objects (moon, cloud). These were designed to elicit both “yes” and “no” responses to ownership questions, and children who had a bias to respond with all “Yes” or all “No” answers were excluded from analyses (*N* = 14). However, the pattern of responses discussed below remains consistent if these children are included.

Children then answered sixteen test questions about living entities in a 2 × 2 design varying ontological type (Human; Animal) and autonomy (Human: Adult/Child; Animal: Wild/Domestic); across trials, there were four entities of each type. The human trials consisted of full body images of equal numbers of male and female adults and children. The wild animals included a giraffe, owl, zebra, and deer, and the domestic animals included a cat, dog, goldfish, and hamster. Trials were presented in one of four randomized orders. For each trial, children were asked “So Henry wants to know—does anyone own this ______?”. Adult participants followed the same procedure, but answered questions using an online survey platform, and were not required to name something they owned.

### Results and Discussion

#### Overview.

**Figure 1.**
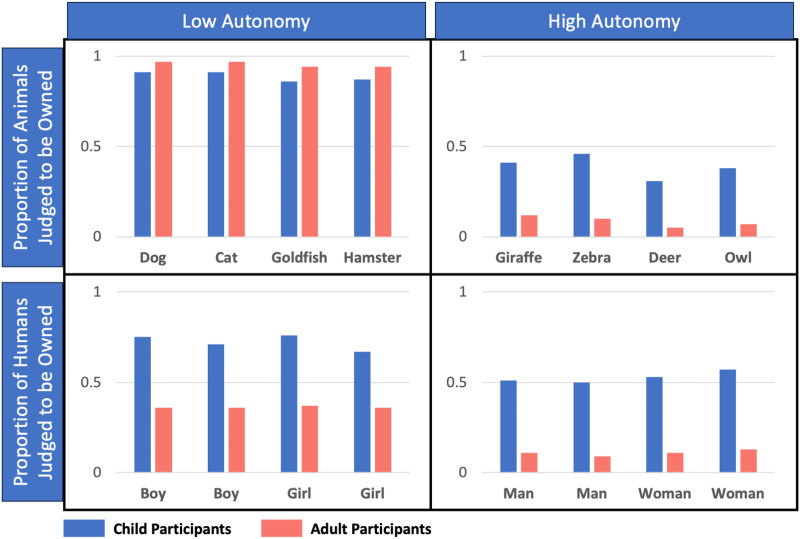
Experiment 1. Proportion of trials in which participants judged that each animal or human was owned. Error bars represent 95% confidence intervals.

There were three main findings. First, both children and adults mostly judged that low-autonomy (domestic) animals were owned, but wild (high-autonomy) animals were not. Second, both children and adults mostly judged it was more likely to own a human child than a human adult. Third, unlike adults, children mostly viewed human children as owned. Judgments for individual entities are depicted in Figure 1.

#### Statistical Approach.

Because each participant contributed a dichotomous response on sixteen consecutive trials, we conducted a binary logistic Generalized Estimating Equation (GEE) to examine ownership judgments, with Autonomy (high/low) and Ontological Type (human/animal) as within-subjects factors and Age Group (adult/child) as a between-subjects factor. This resulted in a significant 3-way interaction between Autonomy, Species, and Age Group, *χ*^2^(1) = 14.67, *p* < .001. To explore this further, we analyzed ownership judgments by children and adults separately. We analyzed responses from each group using a binary logistic GEE with Autonomy (high/low) and Ontological Type (human/animal) as within-subjects factors. To investigate whether children’s judgments changed with age, we also included Age (mean-centered months) as a continuous predictor.

#### Ownership Judgments by Adult Participants.

**Figure 2.**
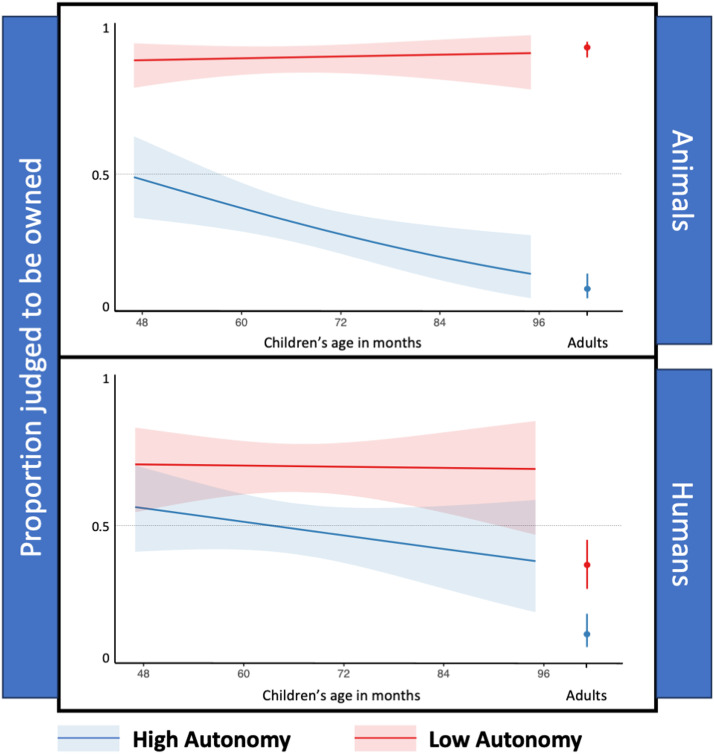
Experiment 1. Proportion of trials in which participants judged that animals (wild, domesticated) and humans (adults, children) were owned. Shaded area and error bars represent 95% confidence intervals.

Adults’ judgments showed a main effect of autonomy, *χ*^2^(1) = 128.73, *p* < .001, as well as a main effect of ontological type, *χ*^2^(1) = 37.95, *p* < .001, and an interaction between autonomy and ontological type, *χ*^2^(1) = 75.53, *p* < .001, see Figure 2.

When judging whether an animal was owned, adults were more likely to judge that low-autonomy (domestic) animals were owned than that high-autonomy (wild) animals were owned, *χ*^2^(1) = 142.00, *p* < .001. Despite alternate possible assessments (e.g., typically wild animals might have been owned by a zoo; typically domestic animals might have been feral), these judgements were nearly unanimous, with adults judging that wild animals were owned only 9% of the time, *χ*^2^(1) = 68.59, *p* < .001, and that domestic animals were owned 96% of the time, *χ*^2^(1) = 90.26, *p* < .001.

When considering humans, adult participants mostly judged that both adults (11%, *χ*^2^(1) = 47.55, *p* < .001) and children (35%, *χ*^2^(1) = 8.10, *p* = .004) were not owned. Interestingly, though, autonomy affected these judgments as well: Adults were more likely to judge that a child was owned than that an adult was owned, *χ*^2^(1) = 28.29, *p* < .001.

#### Ownership Judgments by Child Participants.

Children’s judgments also showed a main effect of autonomy, *χ*^2^(1) = 154.47, *p* < .001, and an interaction between autonomy and ontological type, *χ*^2^(1) = 35.20, *p* < .001, although, perhaps surprisingly, no main effect of ontological type, *χ*^2^(1) = 2.51, *p* = .113, see Figures 1 and 2.

Like adults, children’s judgments about whether an animal was owned depended on whether the animal was autonomous. Children were more likely to judge that domestic (low-autonomy) animals were owned than wild (high-autonomy) animals, *χ*^2^(1) = 116.76, *p* < .001. Children judged that domestic animals were owned on 88% of trials, *χ*^2^(1) = 87.14, *p* < .001, and that wild animals were not owned on 66% of trials, *χ*^2^(1) = 19.53, *p* < .001.

When considering humans, children’s judgments also depended on autonomy. Children were more likely to judge that children (low-autonomy) were owned than that adults (high-autonomy) were owned, *χ*^2^(1) = 29.15, *p* < .001. However, unlike adults, children largely judged that children were owned (69%, *χ*^2^(1) = 18.28, *p* < .001), and responded at chance regarding adults (48%, *χ*^2^(1) = .15, *p* = .696).

The interaction between autonomy and ontological type resulted because although children were more likely to affirm ownership for low-autonomy entities than autonomous ones, this effect was bigger when the entities were animals, *M*_difference_ = 2.75, *SE* = 0.25, *p* < .001, rather than humans, *M*_difference_ = 0.89, *SE* = 0.17, *p* < .001.

#### Age Effects.

We did not observe a main effect of children’s age, *χ*^2^(1) = 1.63, *p* = .202, but age interacted with autonomy, *χ*^2^(1) = 6.18, *p* = .013, such that while children’s judgments about the ownership of low-autonomy entities did not significantly vary with age, *p* = .958, older children were more likely than younger ones to deny that high-autonomy entities were owned, *p* = .015; see Figure 2.

Both adults’ and children’s ownership judgments were affected by whether entities were generally autonomous or non-autonomous. Although adults viewed human children as more ownable than human adults, the majority nonetheless judged all humans as unowned. Children of all ages, however, mostly viewed children as owned. Do children judge that children are specifically owned by their parents, or that children are owned by adults more generally? We explore this in Experiment 2.

## EXPERIMENT 2

In Experiment 1, children mostly judged that children are owned, but they were not asked who they were owned by. Experiment 2 investigated whether children judge that parents own children, or more broadly that children are owned by any adult (such as a teacher). To explore this question, we showed children pairs of adults and children, described as either parent and child or teacher and student. For each pair, children were asked whether the adult owned the child. For comparison, we also included trials asking whether the child owned the adult.

### Method

#### Participants.

We tested 62 children aged 4 to 7 years (52% female, mean age = 75 months). A further 21 children were tested but were excluded for having a “Yes”-bias in their answers to the warmup questions (see below).

#### Procedure.

As in Experiment 1, the experimenter first explained to children that they would be answering questions about owning things, and asked them to name something that they owned. All children provided sensible answers about an item that they owned. Children were then told they would answer some questions about what things people own or don’t own, and answered four warm-up questions designed to elicit both “yes” and “no” responses to questions about the ownership of objects (backpack, hat, school, neighborhood). Children who had a bias to respond with all “Yes” or all “No” answers were excluded from analyses (*N* = 21). The pattern of responses discussed below remains consistent if these children are included, with the exception of age effects as noted below.

Children were then shown full body images of four adult-child pairs that were described as parent-child or teacher-student (e.g., “This is a mother, and this is her son.”). For ease of description, the pair were always opposite gender (e.g., a mother and her son; a teacher and his [female] student). For each pair, children answered whether the adult owned the child (i.e., Does the mother own her son? Does the teacher own his student?), and whether the child owned the adult (i.e., Does the daughter own her father? Does the student own his teacher?). Trials were presented in one of four orders, half of which began with a teacher-student pair, and half of which began with a parent-child pair. Target genders were alternated, so each participant saw a mother and son, father and daughter, male teacher and female student, and female teacher and male student. Between subjects, we counterbalanced which images were referred to as parents/teachers and children/students. Within-subject, we also counterbalanced which side of the screen the child and adult appeared on, whether we identified the child or the adult first, and which ownership question was asked first.

### Results and Discussion

#### Overview.

Children mostly judged that children were owned by their parents, but parents were not owned by children, and teachers and children did not own each other.

#### Statistical Approach.

A binary logistic GEE examined children’s ownership judgments, with target age (child/adult) and relationship (family/school) as within-subject factors, and mean-centered age in months as a continuous predictor.

#### Ownership Judgments.

**Figure 3.**
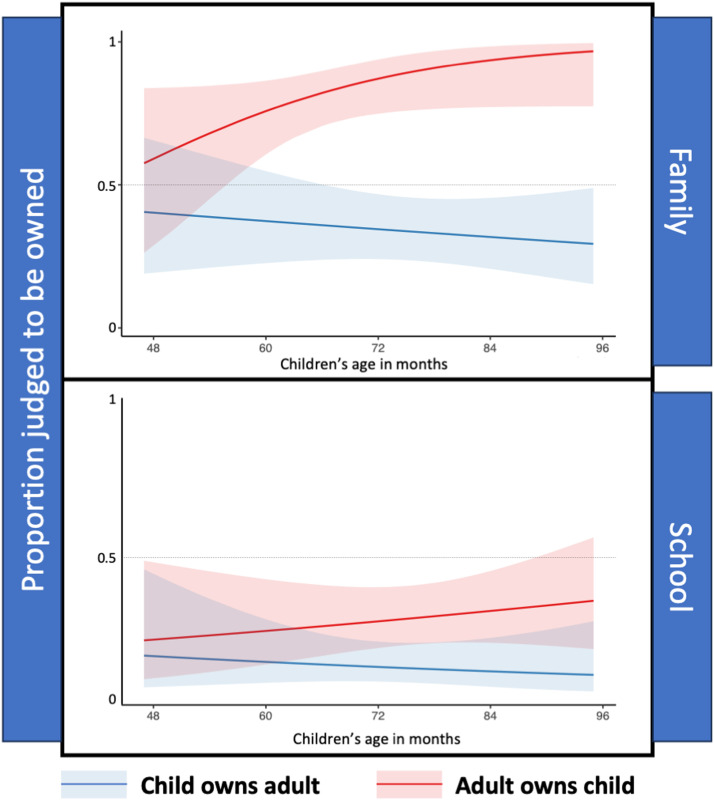
Experiment 2. Proportion of judgments that adults owned children and children owned adults in family and school relationships. Shaded area represents 95% confidence interval.

Children were judged to be owned by adults more often than adults were judged to be owned by children, *χ*^2^(1) = 37.92, *p* < .001. Ownership was also more often affirmed for family relationships than school ones, *χ*^2^(1) = 43.27, *p* < .001. However, these two variables interacted so that target age mattered more for family relationships than for school relationships, *χ*^2^(1) = 8.18, *p* = .004, see Figure 3.

Children were more likely to judge that children were owned by their parent than by their teacher, *χ*^2^(1) = 39.37, *p* < .001. In fact, these judgments differed from chance in opposite directions, with children mostly judging that parents owned children (86% of responses, *χ*^2^(1) = 29.92, *p* < .001), and that teachers did not own students (28% of responses, *χ*^2^(1) = 12.15, *p* < .001). Children’s judgments about whether adults were owned also depended on the relationship between the adult and child. Children were more likely to judge that parents were owned by their children than that teachers were owned by their students, *χ*^2^(1) = 13.98, *p* < .001. However, unlike the judgments about children, participants’ judgments were below chance that both parents (34% of responses, *χ*^2^(1) = 6.97, *p* = .008) and teachers (10% of responses, *χ*^2^(1) = 34.08, *p* < .001) were owned, both *p*s < .001.

#### Age Effects^2^.

There was no main effect of participant age, *χ*^2^(1) = .664, *p* = .415, nor did participant age interact with relationship, *χ*^2^(1) = 1.14, *p* = .287. Participant age interacted with target age, *χ*^2^(1) = 4.88, *p* = .027, though when examined separately, responses for each target age did not significantly depend on participant age: adult owns child, *p* = .066, child owns adult, *p* = .511.

Although both age (i.e., whether the target of ownership was a child or an adult) and relationship (family or school) affected children’s judgments, the only case in which children affirmed ownership at rates above chance was when they were asked whether a parent owns their child. These findings suggest that 4- to 7-year-olds specifically see parents as owning their children. If these judgments are driven by an autonomy principle, then one might ask why most children did not judge that teachers owned children, since teachers also exert control over children. We revisit this question in the General Discussion.

Experiments 1 and 2 found that children mostly view children as owned by their parents, but do not view adults in general, or parents or teachers specifically, as owned. However, there are many differences between children and adults in addition to differences in autonomy. In Experiment 3, we manipulated autonomy directly to investigate whether children judge that children are owned because their parents often decide what they can and cannot do.

## EXPERIMENT 3

To more directly test the role of autonomy in judgments of the ownership of children, we again investigated whether children viewed a parent as owning her child. However, we now manipulated whether the child was depicted as autonomous (lived alone and made all his own decisions) or not (lived with his mother, who made decisions for him).

### Method

#### Participants.

Because children’s age did not substantially impact the findings so far, we collected a smaller age range of children and did not test for effects of age. We tested 40 children aged 5 to 7 years (53% female, mean age = 77 months). A further 8 children were tested but were excluded for having either a “Yes”-bias (4) or a “No”-bias (1) in their answers to the warmup questions (see below), for failing to name an object they owned (1), or for experimenter error (2).

#### Procedure.

As in the previous experiments, all children were first told they would be answering questions about owning things, and were asked to name something that they owned. One child who could not provide a sensible answer about an item that he owned was excluded from analyses. Children then answered five warmup questions designed to elicit both “yes” and “no” responses to ownership questions (backpack, hat, school, neighborhood, cat). Children who had a bias to respond with all “Yes” or all “No” answers (*N* = 5) were excluded from analyses. However, the pattern of responses discussed below remains consistent if these children are included.

**Figure 4.**
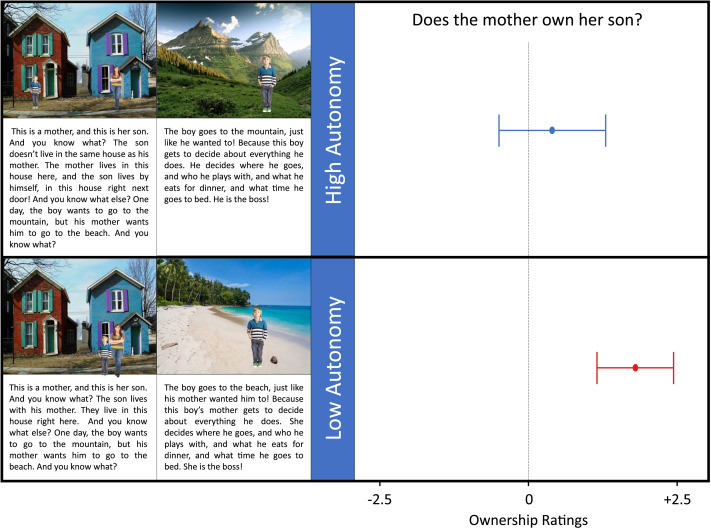
Experiment 3. Stimuli (left) and mean ownership scores (right) for high autonomy and low autonomy (i.e., typical) children. Error bars represent 95% confidence interval.

Children were then shown a photograph depicting a parent, a child, and two houses. Half of the children were assigned to the non-autonomous condition, and were shown the parent and child in front of the same house, and heard a verbal description of a typical, non-autonomous child. The other half of children were assigned to the autonomous condition, and were shown the parent and child in front of separate (but adjacent) houses, and heard a verbal description of an atypical, autonomous child. Figure 4 shows the key images and accompanying verbal script.

Children in both conditions were then asked whether the mother owned her son, and how sure they were (a little, medium, or a lot).

### Results and Discussion

#### Overview.

Children again judged that the typical child was owned by his mother, but were significantly less likely to judge that an autonomous child was owned by his mother.

#### Statistical Approach.

To analyze the results, we multiplied children’s ownership answers (Yes = 1, No = −1) by their confidence ratings (A little = .5, Medium = 1.5, A lot = 2.5), so that each child had a score between −2.5 (very sure the mother does not own her son) and +2.5 (very sure the mother does own her son). We used these values rather than whole numbers so that there was a consistent increase across the entire scale.

#### Ownership Judgments.

Replicating the previous two experiments, children again mostly judged that the non-autonomous (i.e., typical) child was owned by his mother, *M* = 1.80/2.50, *t*(19) = 5.83, *p* < .001. However, children were significantly less likely to judge that the autonomous child was owned by his mother, *M* = .40/2.5, *t*(38) = 2.65, *p* = .012, and these judgments did not differ from chance, *t*(19) = .93, *p* = .362, see Figure 4.

Examining the dichotomous ownership judgments alone, we find a similar pattern, with 89% of children in the non-autonomous condition judging that the mother owned her son, while only 52% of children in the autonomous condition did so, Fisher’s Exact Test, *p* < .001.

Thus, these findings suggest that 5- to 7-year-olds typically view children as owned by their parents. However, if a child is seen as autonomous, with the ability to make his or her own decisions, then children are much less likely to judge that the child is owned.

## GENERAL DISCUSSION

Across three experiments, we found that 4- to 7-year-old children have a robust tendency to think of children as being owned by their parents, but deny that children own parents or that unusually autonomous children are owned by their parents. These judgments are consistent with an autonomy principle, such that highly autonomous entities are less likely to be judged as owned than entities that are low in autonomy.

In Experiment 1, most children taught a puppet that domestic animals and children were owned by somebody, but wild animals and adult humans were not (although they were more sure that wild animals were not owned than that adult humans were not owned). Children’s judgments about animals were consistent with an autonomy-based theory about which living beings are owned—that is, they viewed highly autonomous wild animals as unowned, and low-autonomy domestic animals as owned. It is possible that children’s judgments about animals were influenced by statistical observations that animals like dogs are often owned while most giraffes are not—especially since the test question asked if the animals *are* owned, and not whether they *can* be owned (but see Espinosa & Starmans, 2020 for additional evidence that children’s judgments about the ownership of animals are autonomy-based). But by itself, this kind of statistical account cannot readily explain children’s judgments about the ownership of humans because—from the perspective of most adults, at least—it’s not true that most children are owned.

Children’s judgments of whether humans were owned were also consistent with an autonomy-based theory—they were very likely to say that children were owned, but were at chance when asked about adults. Conversely, the majority of adults denied that any humans were owned, however, as in previous research (Starmans & Friedman, 2016), they were still more likely to judge that children were owned than that adults were. This suggests that although adults may reason about the ownership of living beings based in part on whether they are autonomous, there are either additional factors that override this judgment (e.g., a principle that no human can be owned), or they may simply be unwilling to explicitly say that children are owned. Children, however, generally showed no such discomfort with this view.

In Experiment 2, children mostly judged that parents own children, but children do not own parents, and teachers and students do not own each other. This suggests that children are not confusing family relationships for ownership, nor do they see children as being owned by any adult with authority. Instead, they specifically see children as being owned by their parents. The finding that children do not think teachers own children might be surprising, since children lack autonomy relative to teachers, and teachers, like parents, exert control over children’s actions. However, children recognize that parents have ultimate authority over what happens to their children (e.g., Yau et al., 2009), and this may lead them to conclude that only parents should be considered owners. Also, children may understand that ownership is often exclusive—that is, if something is owned by one person or group, that means it is not (typically) owned by another. Thus, if children think they are owned by their parents, this may lead them to disagree that they are owned by their teachers.

Finally, in Experiment 3, the majority of children again judged that typical children are owned by their parents, but they were less likely to judge that highly autonomous children who live separately from their parents and make all their own decisions were owned. This is the most direct support for the hypothesis that children’s judgments are guided by an autonomy principle, where the ability to make your own decisions means that you are not owned by anyone else.

### Developmental Differences

In contrast with children, adults in Experiment 1 mostly denied that children were owned. This discrepancy in ownership judgments between children and adults suggests that children’s judgments are unlikely to result of direct teaching. Anecdotally, this was supported by parents observing their children’s responses with surprise, often making sure to mention to the experimenter that they had not taught their children that they were owned! The contrast between children’s responses and those of adults likewise does not fit with the idea that young children’s beliefs about ownership principally depend on learning the social norms of their culture (e.g., Rakoczy & Schmidt, 2013). After all, our participants were tested in a culture in which adults typically deny that any human, including a child, can be owned (Starmans & Friedman, 2016).

Instead, children’s reports that parents own their children may be an innocent by-product of their belief that having autonomy—the capacity to make your own choices—prevents entities from being ownable. Children are not generally granted this capacity, and thus, children reason, they are owned by their parents, perhaps much like domestic animals are viewed as owned. This interpretation of our findings is consistent with the idea that children’s understanding of ownership reflects a naïve theory of ownership (Nancekivell et al., 2019; but see Boyer, 2022 for a different perspective). In sum, children’s judgments may reflect a principled understanding of ownership, whereas adults’ responses may be better explained by combining this autonomy principle with adherence to local social norms.

### What Do Children’s Judgments Mean?

In these studies, children repeatedly affirmed that children are typically owned by their parents. The straightforward interpretation of this claim is that children view parents as having a similar relation to their children as they do to their pets, for example. Pets are living, agentic beings, but are typically thought of as uncontroversially owned. Children may simply be expressing their view that the parent-child relationship shares similar features, and thus should also be thought of as ownership.

However, other interpretations of these judgments might also be possible. One possibility is that children might have interpreted the ownership question loosely or metaphorically, rather than truly using principles of ownership to make their judgments. Our findings are unlikely to reflect a simple linguistic misunderstanding that phrases like “my daughter” imply ownership, since children equally say “my mother” and “my teacher”, and yet they denied that children own parents and teachers. However, it is possible that in affirming that children are owned, children might merely have been trying to communicate that some ownership-like relation holds between children and their parents, but without really attributing ownership. Although previous accounts of word-learning suggested that children should find polysemy (and thus metaphorical senses of a word) to be difficult to acquire (e.g., Markman, 1991; Trueswell et al., 2013; Yu & Smith, 2007), more recent research suggests that this kind of polysemy and metaphorical extension is acquired relaticely easily by children and may even facilitate word-learning (Srinivasan & Rabagliati, 2021). If we take the ownership of physical objects to be the standard case, should we interpret the extension of the term “owns” to other domains—such as ideas, digital objects, copyrights, animals, romantic partners, or even, as here, one’s children—as literal, or metaphorical?

One way of making further headway into this issue might be to investigate children’s judgments of the consequences of being owned for both humans and animals. Children as young as ages 3 and 4 understand that owners can use their objects, exclude other people from using them, and give them away (for a review see Pesowski et al., 2022). The consequences of owning a living being likely differ; pets and other animals have some rights that objects do not have, and so owners can’t simply decide to destroy an animal. Nonetheless, if children’s judgments about parents’ rights and responsibilities toward children align closely with their judgments about owners’ rights and responsibilities toward pets, this may suggest that a similar concept is at work in both domains.

This challenge of identifying the bounds of ownership psychology and determining where it begins and ends runs through much work on ownership. With the present research, the concern is that children (and some adults) may use the language of ownership in contexts where the psychology of ownership isn’t operating. But much of the time, the concern runs in the *opposite* direction: Ownership psychology may operate even when people refrain from explicitly attributing ownership, or may underlie language that pretends to be metaphorical (see Wilson & Daly, 1992 for an early discussion, and Boyer, 2022 for an extensive treatment of this issue). This suggests that an alternative way of understanding why children’s judgments differ from those of adults is that adults may conceive of children as owned, even though the majority do not linguistically acknowledge this. This account might also help to explain why a non-trivial number of adults affirmed that children are owned by their parents.

### Additional Principles of Ownership

Although our findings suggest that children’s beliefs that they are owned by their parents stem at least in part from their reasoning about autonomy, these beliefs could also be supported by other principles of ownership. One such principle is the understanding that children are created by their parents (e.g., Johnson & Solomon, 1997), as preschoolers normally judge that things belong to their creators (e.g., Kanngiesser & Hood, 2014; Levene et al., 2015; Rochat et al., 2014; also see Davoodi et al., 2020). Use of this creation principle might help explain why children thought that children belong to their parents rather than to their teachers, despite both parents and teachers having authority over children. However, this account cannot be the full story because use of the creation principle does not explain why children in Experiment 3 were less likely to say the highly autonomous child was owned by his mother. So although the creation principle may contribute to children’s beliefs that children are owned by their parents, it cannot replace the autonomy account.

Another possibility is that children’s belief that they are owned by their parents is shaped by the understanding that children and their parents typically live together. Children and adults often judge that people own entities and other items found on their land (DeScioli & Karpoff, 2015; DeScioli et al., 2017; Espinosa & Starmans, 2020; Goulding & Friedman, 2018) and so this might again help explain why children thought they belong to their parents but not to their teachers. Such territory-based inferences could also have contributed to judgments that the highly autonomous boy in Experiment 3 did not belong to his mother, since he was described as living apart from her.

However, this territory-based account cannot fully explain our findings. For one thing, children deny that people own animals on their property if the animals are free to leave their property (Espinosa & Starmans, 2020). Likewise, children don’t believe that they own their parents, even though they live with them. While territory-based intuitions might play some role in raising one’s belief that someone or something is owned, they must also interact with autonomy-based reasoning, since these prior findings suggest that they apply only to the ownership of objects and beings judged to be low-autonomy.

Thus, autonomy-based reasoning may be necessary to explain the pattern of results observed in the current experiments. However, along with differences in autonomy, there are of course many other differences between children and adults, and wild animals and domestic animals. One fruitful avenue for future research may thus be to assess autonomy judgments directly, and investigate whether these judgments predict whether an entity will be seen as owned.

### Beyond Ownership

Looking beyond the consequences of our findings for accounts of ownership, our results may add to previous findings that there are developmental differences in the tendency to afford humans a privileged moral status in comparison with non-human animals (and other kinds of entities). In our first experiment, adults were reluctant to say that humans of any age were owned (also see Starmans & Friedman, 2016). But children did not seem to feel this discomfort. They did not show a “speciesist” effect, in that they were not overall more likely to view animals as ownable than humans. They also did not show a strong tendency to avoid saying adult humans were owned, answering at chance levels in Experiment 1. Most notably, across all experiments, the majority of children judged that (typical) children are owned by parents. These results fit with other recent findings suggesting that the belief that humans are morally distinct from other kinds of animals may arise relatively late in development (e.g., Caviola et al., 2019; McGuire et al., 2023; Sommer et al., 2019; Wilks et al., 2021).

The differences here between children and adults raise other questions. What happens as children age to make them lose the intuition that children are owned? Is it, as suggested above, the acquisition of a social taboo or a special moral status for humans? Or do adults differ from children in their conception of autonomy, their notion of ownership, or their beliefs about children’s capacities? This leads to a more troubling question: What is going on in the minds of individuals who believe that it is fully possible to own another person, as with the white plantation owners in the Antebellum South? Did they have a different conception of ownership than modern people, or did they view enslaved peoples as, in a very real sense, less than human (Smith, 2021), and thus lacking the key properties of autonomy? Here, as elsewhere, intuitions about autonomy have significant moral importance.

## FUNDING INFORMATION

This research was supported by a Social Sciences and Humanities Research Council Discovery Grant awarded to OF.

## OPEN DATA AND MATERIALS

All data and stimuli are available at: https://osf.io/gxmkd/.

## Notes

^1^ This said, slavery has taken many different forms throughout history and in some of these forms enslaved people appear to have had substantial autonomy. For instance, in some societies, enslaved people could own property and even enslave others. This has led some to suggest that “… the opposite of slavery in most societies (and with the striking exception of the modem West) is some notion not of autonomy, but of citizenship …” (Kopytoff, 1982). An alternative view, though, is that even if enslaved people were sometimes recognized as having some degree of autonomy, this was likely nonetheless quite constrained if they could be compelled to labor against their wishes.^2^ The only instance in which we observed a somewhat different pattern of responses when the excluded children were included was in the age effects in Experiment 2. When including all participants, there was a main effect of participant age, *χ*^2^(1) = 4.36, *p* = .037, and participant age interacted with relationship, *χ*^2^(1) = 11.97, *p* < .001, and target age, *χ*^2^(1) = 14.94, *p* < .001. For family relationships, older children were less likely to judge that children owned parents, *χ*^2^(1) = 8.62, *p* = .003, but equally likely to say that parents owned children, *χ*^2^(1) = 2.67, *p* = .102. For school relationships, older children were less likely to judge either that children owned teachers, *χ*^2^(1) = 14.13, *p* < .001, or that teachers owned children, *χ*^2^(1) = 4.25, *p* = .039.
